# *cis*-Bis­[2-(diphenyl­phosphino)benzene­thiolato-κ^2^ *P*,*S*]palladium(II)

**DOI:** 10.1107/S1600536808026718

**Published:** 2008-08-23

**Authors:** Jaime Fierro-Arias, David Morales-Morales, Simón Hernández-Ortega

**Affiliations:** a Instituto de Química, Universidad Nacional Autónoma de México, Circuito Exterior, Ciudad Universitaria, México 04510, D. F., México.

## Abstract

The title compound, [Pd(C_18_H_14_PS)_2_], was synthesized by the reaction of (Ph_2_PC_6_H_4_SH) with [PdCl_2_(NCC_6_H_5_)_2_] in a 2:1 molar ratio in the presence of a slight excess of NEt_3 _as base in dichloro­methane. The compound crystallizes with the Pd(II) atom on a twofold rotation axis. The palladium center has a slightly distorted square-planar environment, with the two P—S chelating ligands adopting a *cis* configuration. The present structure is a pseudo-polymorph of [Pd(C_18_H_14_PS)_2_]·CH_2_Cl_2_.

## Related literature

For related literature, see: Andreasen *et al.* (1999 ▶); Braunstein & Naud (2001 ▶); Real *et al.* (2000 ▶); Canseco-Gonzalez *et al.* (2003 ▶, 2004 ▶); Dilworth & Weatley (2000 ▶); Dilworth *et al.* (2000 ▶); Gómez-Benítez *et al.* (2003 ▶, 2007*a* ▶,*b* ▶); Morales-Morales *et al.* (2002*a* ▶,*b* ▶); Ortner *et al.* (2000 ▶); Ríos-Moreno *et al*. (2005 ▶); Taguchi *et al.* (1999 ▶).
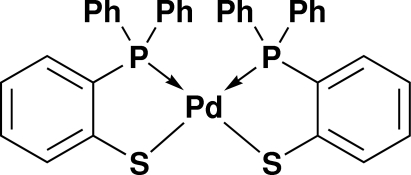


## Experimental

### 

#### Crystal data

[Pd(C_18_H_14_PS)_2_]
*M* *_r_* = 693.04
Trigonal, 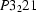

*a* = 9.306 (1) Å
*c* = 30.069 (8) Å
*V* = 2255.2 (7) Å^3^
*Z* = 3
Mo *K*α radiation
μ = 0.89 mm^−1^
*T* = 298 (2) K
0.16 × 0.07 × 0.04 mm

#### Data collection

Bruker SMART APEX CCD area-detector diffractometer
Absorption correction: multi-scan (*SADABS*; Sheldrick, 1996 ▶) *T*_min_ = 0.877, *T*_max_ = 0.967
18737 measured reflections
2749 independent reflections
1811 reflections with *I* > 2σ(*I*)
*R*_int_ = 0.113

#### Refinement

*R*[*F* ^2^ > 2σ(*F* ^2^)] = 0.046
*wR*(*F* ^2^) = 0.084
*S* = 0.82
2749 reflections
186 parameters
H-atom parameters constrained
Δρ_max_ = 1.40 e Å^−3^
Δρ_min_ = −0.31 e Å^−3^
Absolute structure: Flack (1983 ▶), 1113 Friedel Pairs
Flack parameter: −0.05 (6)

### 

Data collection: *SMART* (Bruker, 1999 ▶); cell refinement: *SAINT* (Bruker, 1999 ▶); data reduction: *SAINT*; program(s) used to solve structure: *SHELXTL* (Sheldrick, 2008 ▶); program(s) used to refine structure: *SHELXTL*; molecular graphics: *SHELXTL*; software used to prepare material for publication: *SHELXTL*.

## Supplementary Material

Crystal structure: contains datablocks I. DOI: 10.1107/S1600536808026718/bt2741sup1.cif

Structure factors: contains datablocks I. DOI: 10.1107/S1600536808026718/bt2741Isup2.hkl

Additional supplementary materials:  crystallographic information; 3D view; checkCIF report

## Figures and Tables

**Table:** 

Pd—P1	2.2861 (18)
Pd—S1	2.316 (2)

**Table:** 

P1^i^—Pd—P1	101.33 (9)
P1^i^—Pd—S1	171.41 (7)
P1—Pd—S1	86.90 (7)
S1—Pd—S1^i^	85.00 (11)

Symmetry code: (i) 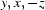.

**Fig. 1.**
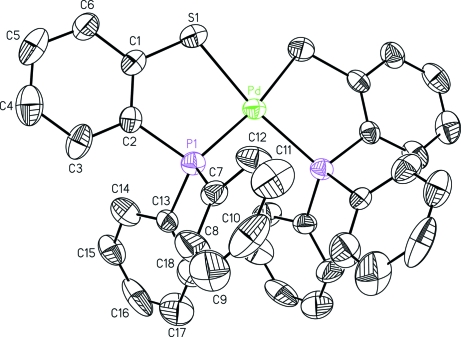
Molecular structure of the title compound with the atoms numbering scheme. Displacement ellipsoids are shown at the 40% probability level. All H atoms have been omitted for clarity. The symmetry operator for generating equivalent atoms symmetry operator; y, x, -z.

## supplementary crystallographic information

### Comment

In recent years, attention has increasingly been paid to the coordination chemistry of polydentate ligands incorporating both thiolate and tertiary phosphine donor ligands, as their combination is likely to confer unusual structures and reactivities on their metal complexes (Dilworth, *et al.*, 2000; Morales-Morales *et al.*, 2002*a*; Gómez-Benítez *et al.*, 2003). These complexes have shown an intriguing variety of structures (Andreasen *et al.* 1999; Taguchi *et al.*, 1999) or unusual oxidation states and enhanced solubility (Ortner *et al.*, 2000), making these species excellent candidates for further studies in reactivity. Moreover, the presence of these ligands in the coordination sphere of transition metal complexes may render interesting behaviours in solution as these ligands can be capable of full or partial de-ligation (hemilability) (Dilworth & Weatley, 2000; Braunstein & Naud, 2001) being able to provide important extra coordination sites for incoming substrates during a catalytic process (Dilworth & Weatley, 2000, Braunstein & Naud, 2001). Thus, given our continuous interest in the synthesis of transition metal complexes bearing P—S hybrid ligands (Morales-Morales *et al.*, 2002*a*, 2002*b*; Gómez-Benítez  *et al.*, 2007*a*, 2007*b*; Ríos-Moreno  *et al.*, 2005; Canseco-Gonzalez *et al.*, 2003, 2004) we   report the crystal structure of a pseudo-polymorph of the Pd(II) complex *cis*- [Pd(Ph_2_PC_6_H_4_-2-S)_2_] (Real *et al.*, 2000; Canseco-Gonzalez *et al.*, 2003)

The asymmetric unit cointains only half of molecule, with the Pd atom in special position of site symmetry 2. The structure of the title compound is shown with the numbering scheme in Fig. 1.   The geometric parameters do not differ significantly from the values reported in the previously described polymorphs. The complex exhibits the Pd center into a slightly distorted square planar environment with the two P—S chelating ligands adopting a *cis* conformation.

### Experimental

Synthesis of *cis*-[Pd(Ph_2_PC_6_H_4_–2-S)_2_] (I). To a solution of (Ph_2_PC_6_H_4_–2-SH) (34 mg, 0.12 mmol) in CH_2_Cl_2_ (20 ml), NEt_3_ (13 mg, 0.13 mmol) and a CH_2_Cl_2_ solution (30 ml) of [Pd(Cl)_2_(NCC_6_H_5_)_2_] (22 mg, 0.058 mmol) were added under stirring. The resulting mixture was allowed to stir overnight. After this time, formation of a pale yellow precipitated was noticed, the product was filtered off under vacuum and washed twice with methanol. Recrystallization from a double layer solvent system CH_2_Cl_2_/MeOH afforded complex I as a microcrystalline yellow powder. Yield 87.5%. ^1^*H*-NMR (300 MHz, CDCl_3_), (7.71–6.79 (m, Ph, 28H); ^31^P-NMR (121 MHz, CDCl_3_), (-42.42 (*s*). Elem. Anal. Calculated for [C_36_H_28_P_2_S_2_Pd] Calc. %: C: 64.20, H: 4.00. Found %: C: 64.00, H: 4.20. MS-FAB^+^ [*M*^+^] = 692 m/*z*.

### Refinement

H atoms were included in calculated positions (C—H = 0.93 Å), and refined using a riding model,with *U*_iso_(H) = 1.2*U*_eq_ of the carrier atom. Geometrical restraints were applied in phenyl rings on P atom.

### Crystal data

**Table:**

[Pd(C_18_H_14_PS)_2_]	*Z* = 3
*M**_r_* = 693.04	*F*_000_ = 1056
Trigonal, *P*3_2_2_1_	*D*_x_ = 1.531 Mg m^−^^3^
Hall symbol: P 32 2"	Mo *K*α radiation λ = 0.71073 Å
*a* = 9.306 (1) Å	Cell parameters from 2935 reflections
*b* = 9.306 (1) Å	θ = 2.5–19.9º
*c* = 30.069 (8) Å	µ = 0.89 mm^−^^1^
α = 90º	*T* = 298 (2) K
β = 90º	Prism, yellow
γ = 120º	0.16 × 0.07 × 0.04 mm
*V* = 2255.2 (7) Å^3^	

### Data collection

**Table:**

Bruker SMART APEX CCD area-detector diffractometer	2749 independent reflections
Radiation source: fine-focus sealed tube	1811 reflections with *I* > 2σ(*I*)
Monochromator: graphite	*R*_int_ = 0.113
Detector resolution: 0.83 pixels mm^-1^	θ_max_ = 25.3º
*T* = 298(2) K	θ_min_ = 2.0º
ω scans	*h* = −11→11
Absorption correction: multi-scan(SADABS; Sheldrick, 1996)	*k* = −11→11
*T*_min_ = 0.877, *T*_max_ = 0.967	*l* = −36→36
18737 measured reflections	

### Refinement

**Table:**

Refinement on *F*^2^	Hydrogen site location: inferred from neighbouring sites
Least-squares matrix: full	H-atom parameters constrained
*R*[*F*^2^ > 2σ(*F*^2^)] = 0.046	*w* = 1/[σ^2^(*F*_o_^2^) + (0.025*P*)^2^] where *P* = (*F*_o_^2^ + 2*F*_c_^2^)/3
*wR*(*F*^2^) = 0.084	(Δ/σ)_max_ = 0.001
*S* = 0.82	Δρ_max_ = 1.40 e Å^−^^3^
2749 reflections	Δρ_min_ = −0.31 e Å^−^^3^
186 parameters	Extinction correction: none
Primary atom site location: structure-invariant direct methods	Absolute structure: Flack (1983), 1113 Friedel Pairs
Secondary atom site location: difference Fourier map	Flack parameter: −0.05 (6)

### Special details

**Table:**

Geometry. All e.s.d.'s (except the e.s.d. in the dihedral angle between two l.s. planes) are estimated using the full covariance matrix. The cell e.s.d.'s are taken into account individually in the estimation of e.s.d.'s in distances, angles and torsion angles; correlations between e.s.d.'s in cell parameters are only used when they are defined by crystal symmetry. An approximate (isotropic) treatment of cell e.s.d.'s is used for estimating e.s.d.'s involving l.s. planes.
Refinement. Refinement of *F*^2^ against ALL reflections. The weighted *R*-factor *wR* and goodness of fit *S* are based on *F*^2^, conventional *R*-factors *R* are based on *F*, with *F* set to zero for negative *F*^2^. The threshold expression of *F*^2^ > σ(*F*^2^) is used only for calculating *R*-factors(gt) *etc*. and is not relevant to the choice of reflections for refinement. *R*-factors based on *F*^2^ are statistically about twice as large as those based on *F*, and *R*- factors based on ALL data will be even larger.

### Fractional atomic coordinates and isotropic or equivalent isotropic displacement parameters (Å^2^)

**Table:**

	*x*	*y*	*z*	*U*_iso_*/*U*_eq_	
Pd	0.48559 (7)	0.48559 (7)	0.0000	0.0377 (2)	
P1	0.2993 (2)	0.3605 (2)	0.05647 (6)	0.0399 (5)	
S1	0.6481 (2)	0.6901 (2)	0.05082 (7)	0.0592 (6)	
C1	0.5327 (8)	0.6409 (8)	0.0997 (2)	0.0408 (18)	
C2	0.3770 (9)	0.4982 (8)	0.1037 (2)	0.0397 (18)	
C3	0.2892 (10)	0.4667 (9)	0.1433 (2)	0.060 (2)	
H3	0.1854	0.3719	0.1458	0.072*	
C4	0.3528 (12)	0.5730 (12)	0.1790 (2)	0.072 (3)	
H4	0.2918	0.5509	0.2051	0.086*	
C5	0.5077 (12)	0.7126 (11)	0.1757 (3)	0.069 (3)	
H5	0.5526	0.7837	0.1999	0.083*	
C6	0.5958 (10)	0.7467 (9)	0.1367 (2)	0.059 (2)	
H6	0.6995	0.8419	0.1347	0.070*	
C7	0.2670 (9)	0.1641 (8)	0.0782 (2)	0.0457 (19)	
C8	0.1239 (11)	0.0432 (10)	0.0973 (3)	0.076 (3)	
H8	0.0317	0.0570	0.0987	0.091*	
C9	0.1143 (15)	−0.0996 (12)	0.1145 (3)	0.095 (3)	
H9	0.0142	−0.1808	0.1265	0.114*	
C10	0.2415 (15)	−0.1239 (12)	0.1144 (3)	0.083 (3)	
H10	0.2336	−0.2182	0.1275	0.100*	
C11	0.3848 (12)	−0.0089 (12)	0.0949 (3)	0.085 (3)	
H11	0.4754	−0.0254	0.0939	0.102*	
C12	0.3967 (11)	0.1320 (11)	0.0765 (2)	0.064 (2)	
H12	0.4949	0.2079	0.0625	0.077*	
C13	0.0948 (8)	0.3330 (8)	0.0457 (2)	0.0414 (19)	
C14	0.0640 (11)	0.4611 (12)	0.0535 (2)	0.064 (2)	
H14	0.1450	0.5570	0.0673	0.076*	
C15	−0.0844 (10)	0.4507 (12)	0.0413 (3)	0.065 (3)	
H15	−0.1031	0.5386	0.0465	0.078*	
C16	−0.2044 (12)	0.3062 (16)	0.0212 (3)	0.094 (4)	
H16	−0.3033	0.2980	0.0118	0.113*	
C17	−0.1781 (11)	0.1752 (13)	0.0151 (3)	0.105 (3)	
H17	−0.2609	0.0766	0.0029	0.125*	
C18	−0.0297 (10)	0.1907 (11)	0.0272 (3)	0.080 (3)	
H18	−0.0127	0.1014	0.0228	0.096*	

### Atomic displacement parameters (Å^2^)

**Table:**

	*U*^11^	*U*^22^	*U*^33^	*U*^12^	*U*^13^	*U*^23^
Pd	0.0332 (3)	0.0332 (3)	0.0451 (4)	0.0155 (4)	−0.0016 (2)	0.0016 (2)
P1	0.0406 (11)	0.0357 (12)	0.0438 (12)	0.0193 (9)	−0.0009 (9)	0.0022 (9)
S1	0.0476 (13)	0.0509 (13)	0.0634 (14)	0.0129 (10)	−0.0031 (10)	−0.0121 (11)
C1	0.051 (5)	0.038 (4)	0.042 (4)	0.029 (4)	−0.011 (4)	−0.002 (3)
C2	0.050 (5)	0.041 (4)	0.037 (4)	0.029 (4)	−0.002 (4)	0.000 (3)
C3	0.094 (7)	0.061 (5)	0.034 (5)	0.046 (5)	−0.007 (5)	−0.002 (4)
C4	0.105 (8)	0.091 (7)	0.038 (5)	0.064 (7)	0.003 (5)	0.005 (5)
C5	0.105 (8)	0.067 (6)	0.048 (6)	0.053 (6)	−0.025 (5)	−0.019 (5)
C6	0.057 (6)	0.057 (5)	0.062 (5)	0.029 (5)	−0.010 (5)	−0.018 (4)
C7	0.050 (5)	0.035 (5)	0.042 (4)	0.014 (4)	0.003 (3)	0.002 (3)
C8	0.073 (6)	0.046 (6)	0.111 (7)	0.033 (5)	0.040 (5)	0.031 (5)
C9	0.119 (10)	0.055 (7)	0.103 (8)	0.038 (7)	0.040 (8)	0.039 (6)
C10	0.138 (9)	0.044 (6)	0.069 (6)	0.046 (7)	−0.023 (6)	0.000 (5)
C11	0.096 (8)	0.079 (8)	0.100 (8)	0.059 (7)	−0.039 (6)	−0.006 (6)
C12	0.046 (6)	0.064 (6)	0.082 (6)	0.027 (4)	−0.004 (5)	0.011 (5)
C13	0.040 (4)	0.039 (5)	0.040 (4)	0.016 (4)	0.010 (3)	0.003 (3)
C14	0.063 (6)	0.071 (7)	0.060 (5)	0.036 (5)	0.014 (5)	0.023 (5)
C15	0.056 (5)	0.096 (8)	0.065 (6)	0.055 (6)	0.005 (4)	0.012 (5)
C16	0.068 (7)	0.185 (13)	0.059 (6)	0.085 (9)	0.018 (5)	0.027 (7)
C17	0.053 (6)	0.153 (10)	0.119 (7)	0.060 (7)	−0.031 (6)	−0.039 (7)
C18	0.059 (6)	0.095 (8)	0.095 (7)	0.045 (6)	−0.012 (5)	−0.021 (6)

### Geometric parameters (Å, °)

**Table:**

Pd—P1^i^	2.2861 (18)	C8—C9	1.386 (10)
Pd—P1	2.2861 (18)	C8—H8	0.9300
Pd—S1	2.316 (2)	C9—C10	1.312 (10)
Pd—S1^i^	2.316 (2)	C9—H9	0.9300
P1—C2	1.805 (7)	C10—C11	1.358 (11)
P1—C7	1.818 (7)	C10—H10	0.9300
P1—C13	1.818 (7)	C11—C12	1.375 (10)
S1—C1	1.740 (7)	C11—H11	0.9300
C1—C2	1.398 (8)	C12—H12	0.9300
C1—C6	1.405 (8)	C13—C18	1.367 (9)
C2—C3	1.390 (9)	C13—C14	1.379 (9)
C3—C4	1.376 (9)	C14—C15	1.384 (9)
C3—H3	0.9300	C14—H14	0.9300
C4—C5	1.379 (11)	C15—C16	1.386 (11)
C4—H4	0.9300	C15—H15	0.9300
C5—C6	1.374 (9)	C16—C17	1.369 (12)
C5—H5	0.9300	C16—H16	0.9300
C6—H6	0.9300	C17—C18	1.364 (10)
C7—C8	1.368 (9)	C17—H17	0.9300
C7—C12	1.382 (8)	C18—H18	0.9300
			
P1^i^—Pd—P1	101.33 (9)	C7—C8—C9	121.0 (9)
P1^i^—Pd—S1	171.41 (7)	C7—C8—H8	119.5
P1—Pd—S1	86.90 (7)	C9—C8—H8	119.5
P1^i^—Pd—S1^i^	86.90 (7)	C10—C9—C8	122.2 (11)
P1—Pd—S1^i^	171.41 (7)	C10—C9—H9	118.9
S1—Pd—S1^i^	85.00 (11)	C8—C9—H9	118.9
C2—P1—C7	103.7 (3)	C9—C10—C11	118.7 (11)
C2—P1—C13	105.1 (3)	C9—C10—H10	120.6
C7—P1—C13	106.7 (3)	C11—C10—H10	120.6
C2—P1—Pd	106.9 (2)	C10—C11—C12	120.3 (10)
C7—P1—Pd	118.7 (2)	C10—C11—H11	119.9
C13—P1—Pd	114.4 (2)	C12—C11—H11	119.9
C1—S1—Pd	106.1 (2)	C11—C12—C7	122.0 (9)
C2—C1—C6	117.9 (7)	C11—C12—H12	119.0
C2—C1—S1	122.3 (5)	C7—C12—H12	119.0
C6—C1—S1	119.8 (6)	C18—C13—C14	117.7 (7)
C3—C2—C1	119.7 (6)	C18—C13—P1	122.0 (6)
C3—C2—P1	122.7 (6)	C14—C13—P1	120.2 (6)
C1—C2—P1	117.6 (6)	C13—C14—C15	121.8 (10)
C4—C3—C2	121.3 (8)	C13—C14—H14	119.1
C4—C3—H3	119.3	C15—C14—H14	119.1
C2—C3—H3	119.3	C14—C15—C16	118.3 (10)
C3—C4—C5	119.5 (8)	C14—C15—H15	120.8
C3—C4—H4	120.2	C16—C15—H15	120.8
C5—C4—H4	120.2	C17—C16—C15	120.4 (9)
C6—C5—C4	120.0 (7)	C17—C16—H16	119.8
C6—C5—H5	120.0	C15—C16—H16	119.8
C4—C5—H5	120.0	C18—C17—C16	119.5 (10)
C5—C6—C1	121.5 (7)	C18—C17—H17	120.3
C5—C6—H6	119.2	C16—C17—H17	120.3
C1—C6—H6	119.2	C17—C18—C13	122.2 (8)
C8—C7—C12	115.7 (8)	C17—C18—H18	118.9
C8—C7—P1	125.5 (6)	C13—C18—H18	118.9
C12—C7—P1	118.8 (6)		
			
P1^i^—Pd—P1—C2	−177.7 (2)	S1—C1—C6—C5	179.5 (6)
S1—Pd—P1—C2	4.8 (2)	C2—P1—C7—C8	89.7 (7)
S1^i^—Pd—P1—C2	−14.7 (6)	C13—P1—C7—C8	−21.0 (8)
P1^i^—Pd—P1—C7	65.6 (2)	Pd—P1—C7—C8	−152.0 (6)
S1—Pd—P1—C7	−111.9 (3)	C2—P1—C7—C12	−89.0 (7)
S1^i^—Pd—P1—C7	−131.4 (5)	C13—P1—C7—C12	160.3 (6)
P1^i^—Pd—P1—C13	−61.9 (2)	Pd—P1—C7—C12	29.3 (7)
S1—Pd—P1—C13	120.6 (3)	C12—C7—C8—C9	1.4 (12)
S1^i^—Pd—P1—C13	101.1 (5)	P1—C7—C8—C9	−177.3 (7)
P1^i^—Pd—S1—C1	−167.4 (5)	C7—C8—C9—C10	1.8 (16)
P1—Pd—S1—C1	−4.1 (2)	C8—C9—C10—C11	−3.2 (18)
S1^i^—Pd—S1—C1	173.0 (2)	C9—C10—C11—C12	1.5 (16)
Pd—S1—C1—C2	2.5 (6)	C10—C11—C12—C7	1.8 (14)
Pd—S1—C1—C6	−177.4 (5)	C8—C7—C12—C11	−3.1 (12)
C6—C1—C2—C3	1.0 (10)	P1—C7—C12—C11	175.7 (6)
S1—C1—C2—C3	−178.9 (5)	C2—P1—C13—C18	−150.7 (6)
C6—C1—C2—P1	−178.5 (5)	C7—P1—C13—C18	−40.9 (7)
S1—C1—C2—P1	1.6 (8)	Pd—P1—C13—C18	92.5 (6)
C7—P1—C2—C3	−58.1 (6)	C2—P1—C13—C14	33.4 (6)
C13—P1—C2—C3	53.8 (6)	C7—P1—C13—C14	143.1 (5)
Pd—P1—C2—C3	175.7 (5)	Pd—P1—C13—C14	−83.5 (6)
C7—P1—C2—C1	121.4 (6)	C18—C13—C14—C15	−3.0 (11)
C13—P1—C2—C1	−126.8 (5)	P1—C13—C14—C15	173.1 (6)
Pd—P1—C2—C1	−4.9 (6)	C13—C14—C15—C16	0.7 (12)
C1—C2—C3—C4	−0.3 (11)	C14—C15—C16—C17	2.4 (14)
P1—C2—C3—C4	179.1 (6)	C15—C16—C17—C18	−3.0 (14)
C2—C3—C4—C5	−0.9 (11)	C16—C17—C18—C13	0.5 (14)
C3—C4—C5—C6	1.5 (12)	C14—C13—C18—C17	2.4 (12)
C4—C5—C6—C1	−0.8 (11)	P1—C13—C18—C17	−173.6 (6)
C2—C1—C6—C5	−0.4 (10)		

Symmetry codes: (i) *y*, *x*, −*z*.
